# Eminence of Microbial Products in Cosmetic Industry

**DOI:** 10.1007/s13659-019-0215-0

**Published:** 2019-06-18

**Authors:** Prabhuddha L. Gupta, Mahendrapalsingh Rajput, Tejas Oza, Ujwalkumar Trivedi, Gaurav Sanghvi

**Affiliations:** Department of Microbiology, Marwadi University, Rajkot, 360001 India

**Keywords:** Cosmetics, Microbiology, Biosurfactants, Formulations, Cyclodextrin, Emulsions

## Abstract

**Abstract:**

Cosmetology is the developing branch of science, having direct impact on the society. The cosmetic sector is interested in finding novel biological alternatives which can enhance the product attributes as well as it can substitute chemical compounds. Many of the compounds are having biological origin and are acquire from bacteria, fungi, and algae. A range of biological compounds, like bio-surfactant, vitamins, antioxidants, pigments, enzymes, peptides have promising features and beneficial properties. Moreover, these products can be produced commercially with ease. The review will encompass the importance and use of microbial compounds for new cosmetic formulations as well as products associated with it.

**Graphic Abstract:**

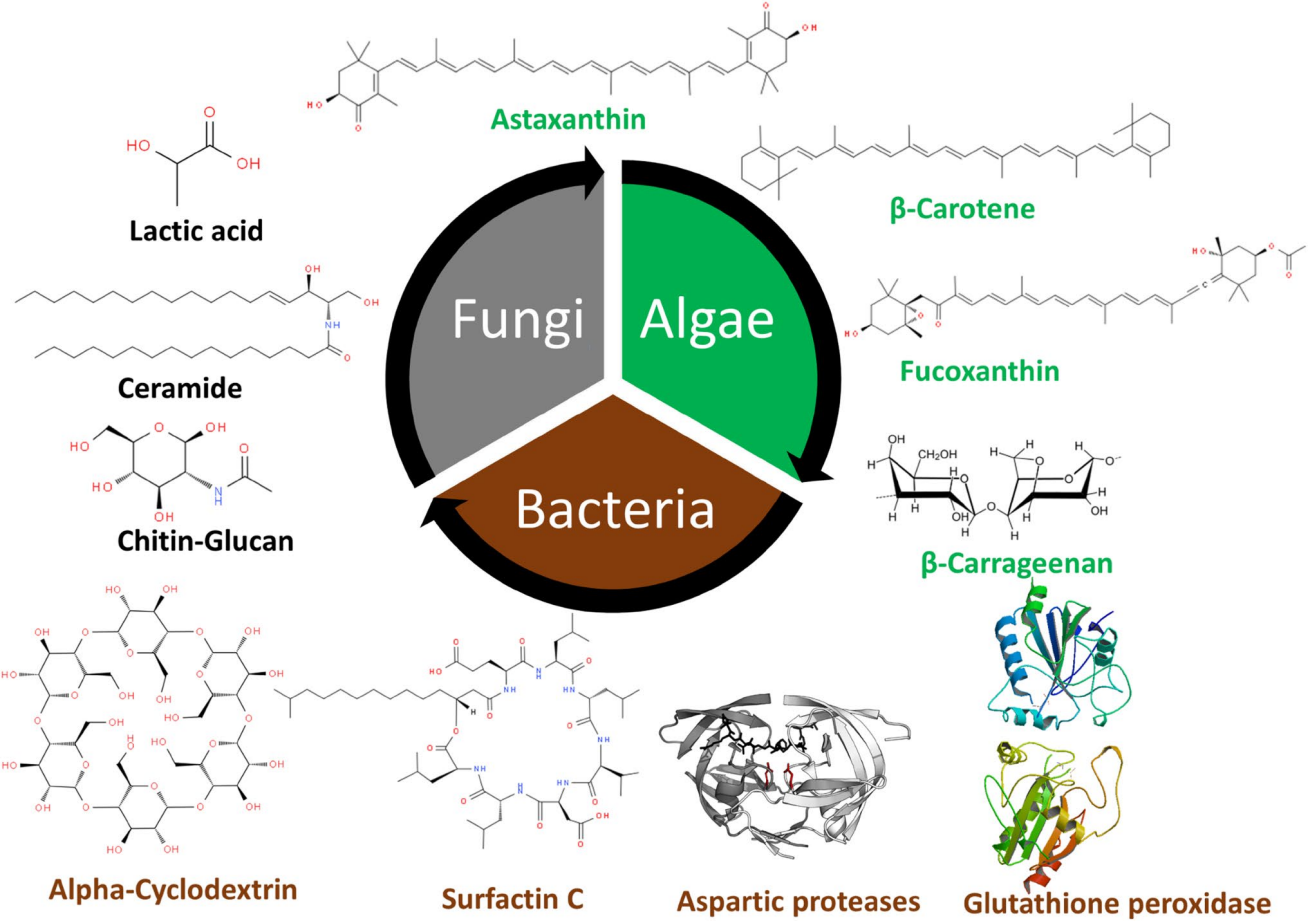

## Introduction

Cosmetics products are the mixture of compounds derived from either biological origin or chemical sources. Cosmetic industry is remarkably prime marketed as over the counter products [1]. However, there are limited guidelines which regulates the full prescription of active ingredients as it is usual cosmetic type formulation [2]. Also, cosmetic products do not provide any monographs governing their formulation and chemicals used, since cosmetic sector is not under stringent control. The US Food, Drug, and Cosmetic Act (FFDCA) in 1938 was first governing body to regulate the key ingredients used in cosmetics industry [3].

Currently, international cosmetics market revenue is estimated to increase up to $429.8 billion by 2022, with compound annual growth rate of 4.3% during the period 2016–2022 (Research and Markets) [4]. The major global cosmetics market is branched into America, Europe and Asia–Pacific. Among Asia–Pacific countries, India represents emerging market for different cosmetic products and has grown rapidly over the last few years. During past decades, India has witnessed a sharp influx of many international brands in the biological derived cosmetic products. Furthermore, the aggressive marketing strategy by the companies, to use ecofriendly and ayurvedic ingredients in cosmetic products have also significantly contributed in elevating the cosmetic market [5]. These biologically derived products have not only found an immense appeal among urban and rural consumers but also in matured population acquainted with the traditional herbal and ayurvedic ingredients [6]. As per the statistic given by Confederation of Indian Industries (CII), currently Indian cosmetic industry is approximately 600 million US dollar and is expected to grow by 15–20% annually.

Cosmetic products are, combination of chemicals, generally used to augment the appearance or odor of the human body [7]. These products are mainly sold at retail counters including super marts, exclusive brand outlets, mouth to mouth and specialty stores. There is a considerable increase in demand for cosmetics over last few decades owing to the development of third world economy and improved standard of living and specialty stores. Furthermore, a shifting preference of beauty products from synthetic to biologically derived sources also have significantly fostered the progress of cosmetics market [8]. In order to withstand and retain market position, cosmetic manufacturing companies are implementing various strategies for identification of biological derived products. The trend to use cosmetics from biologically origin satisfies the upcoming demand of consumers with possible effectiveness [9]. Moreover, the use of herbal cosmetics and organic ingredients from different biological sources have drastically reduced the chances of any possible side effects and hazards of the products. This ultimately, have increased the usage of biological products in cosmetic market. Various strategies are used to develop cosmetic formulations, by involving usage of compounds from bacterial, fungal and algal origin.

The current review represents the role of various microbial compounds having application in cosmetic industry. Various compounds including enzymes and metabolites, originating from diverse microbial sources, and their role in cosmetic industry has been discussed.

## Microbes: An Unveiled Treasure of Novel Compounds

**Fig. 1:**
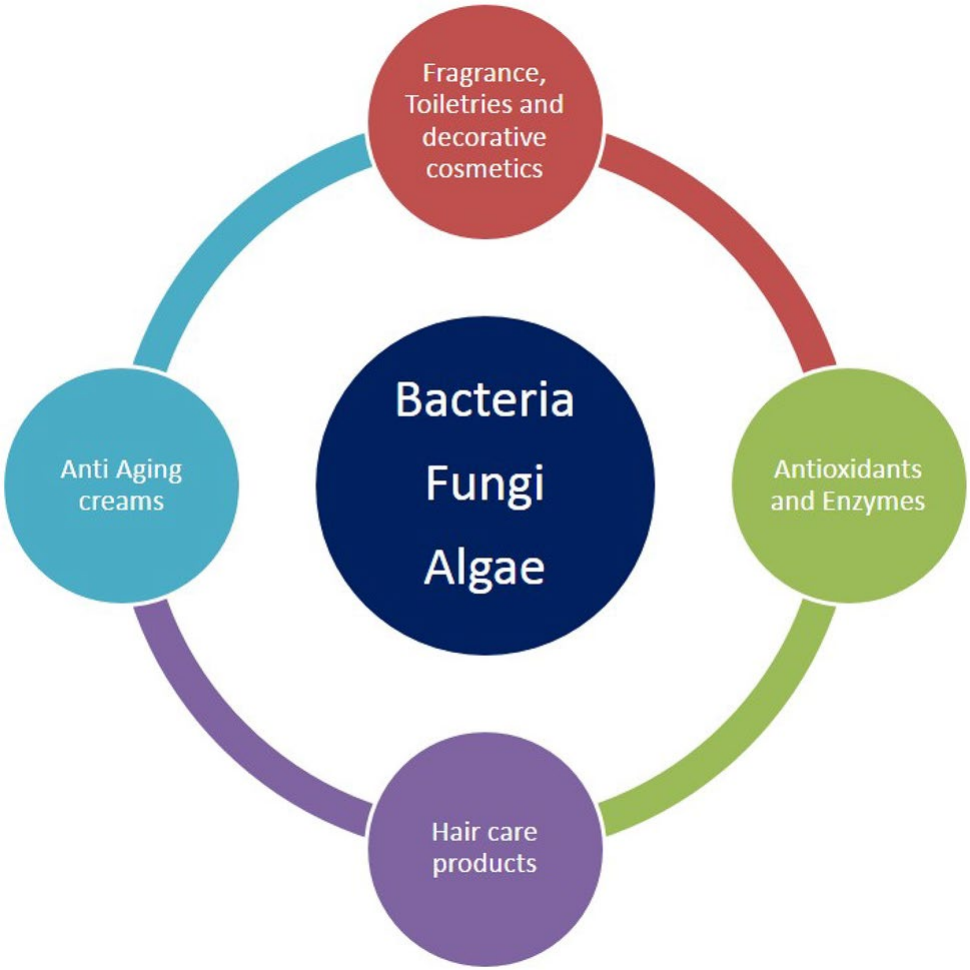
Main categories of cosmetics

Traditionally, biological derived ingredients from plants and various other organism remain a prominent source of novel compounds. Among the diverse sources exists on life, microbes represents one of the cheapest, renewable and novel sources for any chemicals [10]. The omni-present microbial diversity on earth reflects the largest group adapted under different predominant physicochemical conditions [11]. Microbes exist over range of natural habitats including from soil, extreme environments, oceans, glaciers and ponds. The diversity within communities of bacteria, actinomycetes, archaea, lichens, and fungi represents the richness of compounds present in them (Fig. 1). The microbial community represent 30 phylum, majority of which are unculturable and found in different environments [12]. Even though, there is a presence of diversity of microbes in nature, very few of microbes are commercially utilized in cosmetics industry. Hence, the abundantly present but underutilized biodiversity represent a potential opportunity for future biotechnology and cosmetic application [13].

**Fig. 2:**
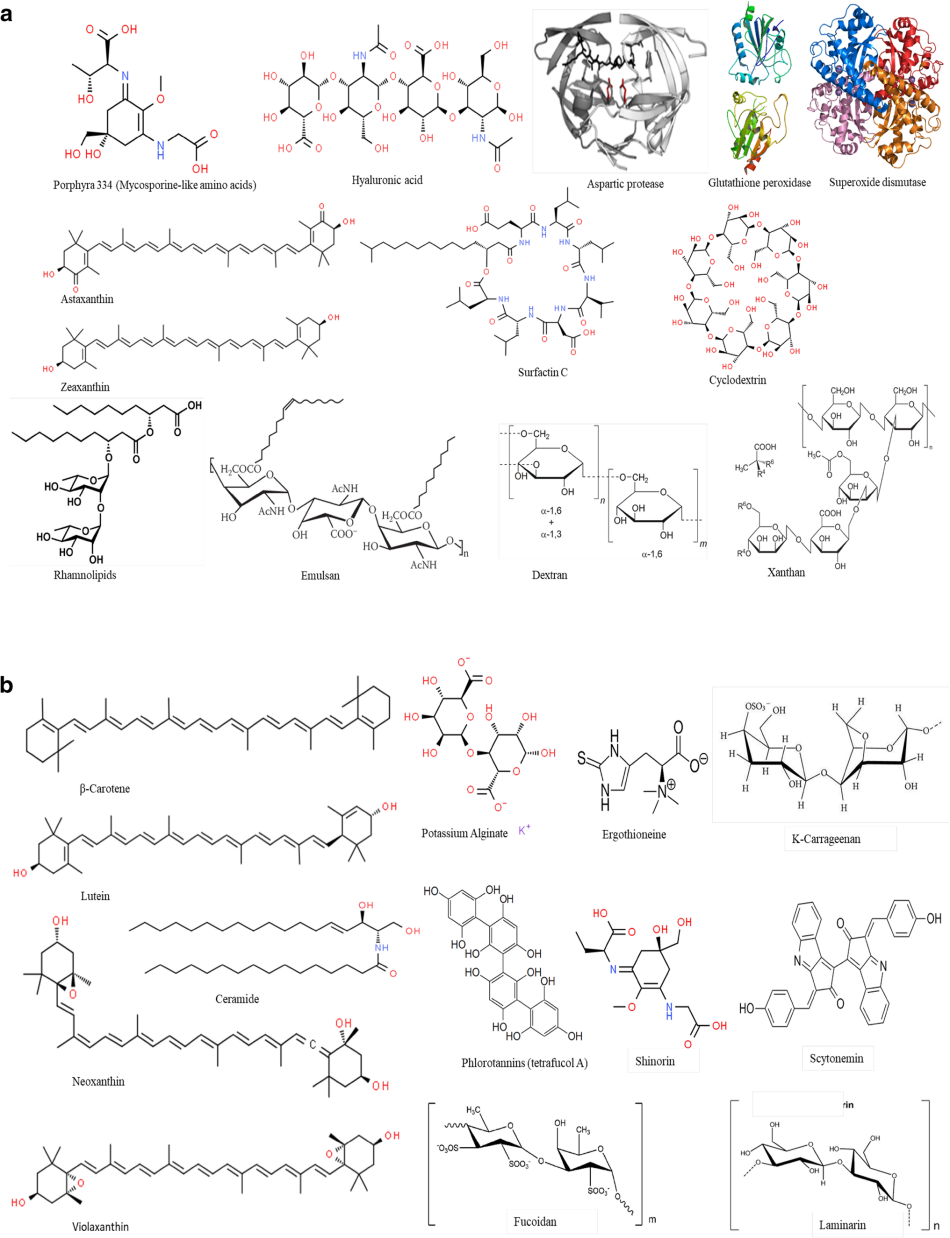
Key structures of various compounds derived from microbial, fungal and algal sources used in cosmetics industry

Studying the chemistry of biological active compounds, isolated from microbes, has tremendously accelerated in recent years. Primarily, the growing demand for bio-molecules is due to their prospective efficacy in cosmetics, drugs, fine chemicals and functional personal-care products [14]. Microorganisms are favorable resources since production of metabolites from microbes is feasible and scale up can be achieved in large quantities with reasonable cost [15]. Moreover, the microbes have ability to adapt and survive distinct conditions that differ from other habitats and accumulate unique bioactive compounds which is not found in other organisms. Microbes are rich in fatty acids, enzymes, peptides, vitamins, lipopolysaccharides and pigments with beneficial properties for cosmetic applications [16] (Fig. 2). Furthermore, unique compound such ceramides, mycosporine-like amino acids carotenoids, and fatty acids such as omega-3, 6, and 9, are obtained from microbes having enormous application in cosmetic industry [17]  (Fig. 2). Increasing consumer demand for biological ingredients and cosmetic products have forced cosmetics industry to explore microbial source [18]. Therefore, the development of new active ingredients for cosmetics products and exploration of biodiversity for such active ingredients have engaged cosmetic companies to protect biodiversity and capitalize market potential and gain competitive advantage.

## Bacteria and Associated Use in Cosmetic Products

Biological compounds, besides medical, pharmaceuticals and food industries, have mottled application in cosmetic industries. Many biological molecules, directly or indirectly, have found key role in the production of various compounds, like esters, aroma compounds and active agents, far and wide used in cosmetic industries. The major advantage of using microbial ingredients is its biocompatibility; additionally, they do have other benefits like simplified process, improved and consistent quality of product and environmental footprint. Of several, microbes, bacteria secrets copious biologically active compounds with significant commercial values; to mention few Oligosaccharides, Exopolysaccharides (EPS), Biosurfactants, Enzymes, Peptides, Vitamins etc. (Table 1). These compounds, replacing chemical compounds, found their application in various cosmetic products used either for beautification or for improving health of the target.

**Table 1:** The bacterial and fungal bio-molecules, source, origin and applications explored in this review

Molecule class	Cosmetic application	Compound	Species	Origin	References
Secondary metabolites	UV and Photo-protective potential, protection from oxidative damage	Mycosporine-like amino acids (MAAs)	*Pseudonocardia* sp. *Actinosynnema mirum* *Streptomyces avermitilis* *Streptomyces lividans* *Corynebacterium glutamicum* *Aurantiochytrium* sp.	Actinomycetes (Actinobacteria)	[53]
Pigments	Improves skin, Antioxidant	Astaxanthin	*Paracoccus*, *Agrobacterium* *aurantiacum*	Bacteria	[54]
			*Thraustochytrids*, *Rhodotorula,* *Phaffia rhodozyma*	Fungi	[55, 56]
	UV protectant, antioxidant, skin hydration	Zeaxanthin	*Corynebacterium autotrophicum*	Bacteria	[57, 58]
Exopolysaccharide	Brightening agent, smoothing agent	Dextran	*L. mesenteriodes*, *Streptococcus mutans*	Bacteria	[10, 33–37, 50–52, 59]
	Moisture retainer, Gelling agent	Alginate	*P. aeruginosa and A. vinelandii*		
	Thickening, gelling, emulsification	Xanthan	*Xanthomonas* spp.		
	Gelling and thickening	Glucuronan	*Sinorhizobium meliloti M5N1CS* and *Gluconacetobacter hansenii*		
	Skin repair, Anti-aging	Hyaluronic acid	*Streptococcus thermophilus,*		
Biosurfactants	Detergent, foaming, emulsifying agent and skin hydrating properties	Viscosin, Rhamnolipids	*Pseudomonas* sp.	Bacteria	[26, 60]
		Mannosylerythritol lipid	*Pseudozyma* sp., *Ustilago* sp., *Candida antartica,*	Fungi	[30, 61]
		Surfactin	*Bacillus subtilis*, *Bacillus pumilus A*, *B. licheniformis and B. amyloliquefacien*	Bacteria	[29, 62–64]
		Emulsan	*Acinetobacter calcoaceticus*	Bacteria	[26]
		Sophorolipid	*Candida* sp.	Fungi	[65]
Cyclodextrins	Sustainable release of aroma, reduce foaming	alpha-cyclodextrin, beta-cyclodextrin, gamma-cyclodextrin	*Bacillus subtilis* Strain 313, *Brevibacterium* sp. Strain 9605, *Brevibacillus brevis* Strain CD 162, *Microbacterium terrae* KNR 9	Bacteria	[19–22, 25]
Enzymes	Treat stretch marks, Scar tissues	Aspartic proteases	*Aspergillus*, *Penicillium*, *Rhizopus*, *Mucor*, *Humicola*, *Thermoascus*, *Thermomyce*, *Conidiobolus coronatus*	Fungi	[41]
	Skin regeneration	Collagenases	*Clostridium histolyticum*, *Vibrio alginolyticus*, *Bacillus cerus*	Bacteria	[48, 66]
	Hair Removal	Keratinases	*Microsporum*, *Epidermophyton*, *Trichophyton*, *Chrysosporiu, Scopulariopsis brevicaulis*, *B. subtilis* and *B. licheniform*	Bacteria	[44, 45, 67]
	Antioxidants, free radical scavenging, anti-aging	Superoxide dismutase Catalases Glutathion peroxidase	*Sulfolobus acidocaldarius, Marinomona*, *Thermus thermophilus*	Bacteria	[39, 40, 68, 69]

### Oligosaccharides

Cyclodextrins, are a group of compounds made up of cyclic oligosaccharides with α-(1,4) linked glucopyranose moiety bound together in a ring and have noteworthy contribution in cosmetic formulations [19]. Cyclodextrin, majorly, is used to reduce the volatility of esters in perfumes and room freshener gels. They also are used abundantly in detergents for steady and sustain release of aroma, thus leading to long lasting effect [20]. Cyclodextrin powders, of smaller size, are used as odor control in talcum, diapers, menstrual discs, pads, napkins, etc. The commercial production of cyclodextrin is more popular by enzymatic transformation rather than chemical synthesis. Therefore, the production of cyclodextrinase enzyme has been extensively carried out by using bacteria strains. *Bacillus subtilis* Strain 313, *Brevibacterium* sp. Strain 9605, *Brevibacillus brevis* Strain CD 162, *Microbacterium terrae* KNR 9 are some of the prominent strains used for the production of cyclodextrin [21–24]. Cyclodextrin glucanotransferase (EC 2.4.1.19) obtained from alkalophilic *Bacillus agaradhaerensis* is a widely sought enzyme in the potential cosmetic preparations [25].

### Biosurfactants

Besides cyclodextrin, biosurfactants are used in preparation of various cosmetic products, owing to the multi-functional property such as detergent, foaming, emulsifying agent and skin hydrating properties. Moreover, biosurfactants are relative non-toxic, and are bio-degradable. In nature, biosurfactants are widely produced by bacteria followed by fungi and other microbes. Most of the biosurfactants belong to fatty acids, neutral lipids, glycolipids and lipopeptides. Furthermore, biosurfactants such as rhamnolipids, are approved by US EPA as safe for use in food products, cosmetics, and pharmaceuticals [26]. One of the most prominently used biosurfactant is Mannosylerythritol lipid (MEL). The basidiomycetous yeast of *Pseudozyma* sps (*P. antarctica, P. aphidis, P.rugulosa, and P. parantarctica*) are well known to produce appreciable amount of MEL. MEL are widely used in the production of various cosmetic products such as lipsticks, lipmakers, eye shades, soap, sprays, powders, nail care, body massage oils and accessories [27, 28]. Another major application for biosurfactants is in anti-wrinkle cosmetics and cleansing products. The use of surfactin derivative lipopeptides has extremely benefited the Japanese cosmetic industry [29]. The most prominent source of surfactin is the *Bacillus sps* (*B. subtilis, B. pumilus A, B. licheniformis and B. amyloliquefaciens*) [30]. Owing to the excellent foaming properties and low CMC (critical micelles concentration) properties, surfactin are extensively used in topically applied dermatological products and in cosmetic formulation of oil and water emulsions [31].

### Exopolysaccharides

The distinctive biocompatibility and non-toxic nature of microbial exopolysaccharides (EPS) has helped considerably to exploit its use in cosmetic industry. Moreover, hydrophilic EPS have high water retention ability which helps to maintain a hydrated environment in skin formulations. One of the very well know EPS produced from glucose polymer is dextran. Dextran is obtained from *Leuconostocaceae* family of microbes such as *Leuconostoc mesenteriodes* and *Streptococcus mutans* [32]. In cosmetics dextran is used as skin smoothing and brightening agent, as it promotes the firmness of skin, promotes radiance, and reduce the appearance of wrinkles. Dextrans also has anti-inflammatory property as it improves the blood flow, augmented nitric oxide (NO) synthesis in the human epidermal keratinocytes cells [33]. Usually alginates are obtained from seaweeds, however bacteria belonging to *Pseudomonas* genera are well known to produce copious amounts alginate as EPS. *Pseudomonas aeruginosa* and *Azotobacter vinelandii* are known to produce alginate which can retain water [34]. Hence, alginate is used as thickner, gelling agent and excipient in skin and cosmetic formulations. The bacterial genus *Xanthomonas* are known to produce complex hetero-polymer EPS known as Xanthan [35]. Xanthan also has thickening properties and assist in gelling hence used in skin formulation to help in skin-smoothing and moisturizing. It also reduces the trans-epidermal water loss in keratinocytes cells. Furthermore, xanthan are also used as emulsifiers and foaming agent in skin formulation [36, 37].

### Proteins, Enzymes and Peptides

In addition to biosurfactants, proteins and peptides also have contributed significantly in the cosmetic industry. Proteins and peptides, since ancient time, are used for improving the quality of the skin, hair or nails. Extensive research has been carried out in the same horizon and identified numerous applications of proteins in the cosmetic industry.

Superoxide dismutase (SOD) and Peroxidase (catalase, glutathione peroxidases, lactoperoxidases) works synergistically as exfoliate. These enzymes serve as scavengers of free radicals and prevent the skin, from the ultra violet light, when applied on the skin surface [38]. Another similar enzyme Lactate dehydrogenase (LDH) capable of catalyzing the reduction of NADH and pyruvate leading to NAD^+^ and lactate as the end product. The above reaction, on the exposure of UV, gets diminished; but, in presence of LDH the subunits remain intact in the cells and allow cell to carry out normal functioning [38]. In past, *Marinomonas* sp., *Sulfolobus acidocaldarius* and few other extremophiles such as *Thermus thermophilus* were used for the production of SOD and/or peroxidase [39]. But, with advances in technology, genetically engineered lactic acid bacteria with high yield and improved stability, have been favorable choice for the production [40].

Proteases are well known enzymes for hydrolyzing the peptide bonds of keratin, collagen and elastin of the skin. Bacterial originated alkaline aspartic proteases from various alkaliphilic bacterial sources have been used to treat skin disorders such as xerosis (dryness of skin), ichthyoses (scaly skin), psoriasis (skin flaking and inflammation) [41]. Furthermore, proteases such as keratinases are known to treat stretch marks, scar tissues and regenerate the epithelial cells to accelerate healing. Commonly, keratin hydrolysates are used in skin topical ointments and creams for heels, knees or elbows which offers external smoothness and reduce damage to the skin. Keratinase is also used in enzymatic peeling treatment, in hair removing and hair growth delaying. *Bacillus licheniformis* has been used commercially for keratinase production commercially exploited organism for keratinase production [42]. Few thermophilic organisms such as *Thermoanaerobacter, Thermosipho,* and *Thermococcus* are also used for production of keratinase [43]. Other Gram-positive bacteria such as *Lysobacter, Nesterenkonia, Kocuria, Microbacterium* and Gram-negative bacteria such as *Vibrio, Xanthomonas, Stenotrophomonas, Chryseobacterium, Fervidobacterium, Thermoanaerobacter,* and *Nesterenkonia* are also potentially known to produce keratinases enzyme [44]. Fungal species of *Microsporum, Epidermophyton, Trichophyton, Chrysosporiu*, are well known keratinophilic fungi and also have potential ability to degrade keratin fibers [45]. Along with enzymes, specific peptides (digest of proteins) have also been widely used in cosmetic preparations. The soluble peptides are used in gels, emulsions, powders and lotions; while, insoluble peptides are used in facial mask [46]. These peptides, for commercial purpose, are generated by controlled action of proteases; which, majorly are secreted from several *Bacillus* spp. [47, 48]. Also, Pentapeptides are widely used for reducing facial wrinkles and roughness [49].

### Hyaluronic Acid

Hyaluronic acid (HA), is a glycosaminoglycan (GAG) consisting of β-4-glucuronic acid (GlcUA) and β-3-N-acetylglucosamine (GlcNAc) [50]. HA is extensively used as dermal filler in cosmetic surgery. Furthermore, many skin lotion, and serums contains sodium hyaluronate as its active ingredient as it boost moisture retention, reduces skin wrinkles and improves skin firmness and elasticity [51]. HA is obtained in large scale from animal tissues from rooster combs. However, microbes belonging to *Streptococcus* genus are also able to synthesize HA. Recently, genetically modified culture of *Bacillus* species are used for the production of HA [52].

## Fungi and Associated Use in Cosmetic Product

Fungi are ambiguous and most diverse organisms. The kingdom Fungi comprises of incredible biodiverse members bridging a comprehensive range of life habitats, life form, size and morphology. The recent high throughput estimates that 5.1 million of fungal species exist in our ecosystem [70]. Numerous potential cosmetics products are developed from fungi for skin care, anti-oxidants and hair products. Among fungi, mushrooms are rich in secondary metabolites known to have various medicinal properties. Compounds such as Schizophyllan derived from *Schizophyllum commune* are known for protective effect of UV rays on skin and benefit in reducing inflammation of skin [71].

### Lactic Acid

Lactic acid is extensively used in cosmetics skin cream to retain skin moisture, impart smoothness and suppleness of skin. High concentration (up to 12%) of lactic acid is used in skin peeling cream as an exfoliating agent, for skin lightening and to reduce acne eruption [72]. Furthermore, poly-l-lactic acid (PLLA) is used as a bio-stimulatory filler and in removal of facial folds, decrease wrinkles and photo-damage [73]. Fungi species of *Rhizopus* genera are known to produce lactic from fermentation of glucose aerobically and has low substrate cost compared to bacterial source such as *Lactobacillus* [74].

### Ceramides

Ceramides is used in cosmetics as skin hydrating agents since the stratum corneum of human epidermal layer contains considerable amount of ceramides. Ceramides are only found in eukaryotic cells and animal origin (e.g. cows). However, concerns regarding infectious diseases have instigated to explore alternative sources to obtain ceramides. Moreover, plant derived ceramides are structurally different from animal ceramides and hence this limits its use in cosmetics. Ceramides from various fungal species have been produced and used in cosmetics [75]. *Candida albicans, Agaricus bisporus, Armillaria tabescens* have used in production of Glycosly ceramides [76]. Furthermore, attempt to obtained ceramides using metabolic engineering in yeast (*Saccharomyces cerevisiae)* also have been developed [77].

### Chitin-glucans

Chitin-glucans are copolymers obtained from the cell wall of fungi and works very well as a good moisturizers [78]. A very renowned example of chitin-glucan is Chitosan. Chitosan are used as antimicrobial agents against dental plaque and readily used in toothpaste formulations [79]. Chitosan along with hyaluronic acid and collagen is also used in hair setting lotions and gels to produce a coating which adds thickness, volume, strength and prevent hair damage. Furthermore, chitosan nanoparticles loaded with minoxidil is used for sustained release of minoxidil for effective transdermal transport and hair growth [80].

### Antioxidants

l-ergothioneine is a powerful anti-oxidant is extracted at high concentrations from mushrooms such as *Portabellas* and *Criminis* species [81]. Owing to the excellent antioxidant properties ergothioneine guards the skin from oxidative and DNA damage and hence it is used in anti-aging creams and lotions [82]. Similarly, Gallic acid is a potentially known to have anti-bacterial and free radical scavenging activities. *Aspergillus niger*, *Fusarium solani* and *Trichoderma viridae* are known to produce gallic acid [83, 84]. Trehalose is another antioxidant compound found in various mushrooms such as *Lentinula edodes*, *Grifola fondosa*, *Pholiota nameko* and *Auricularia auricula*-*judae*. Terahalose has high water retaining capacity and excellent antioxidant property. This makes it useful as in moisturizer creams in cosmetics products.

## Algae and Associated Use in Cosmetic Products

Algae represent an enormous bio-diverse species with more than 72500 reported in phylo–genetic classification [85]. Production of bioactive compounds from algae can be easily manipulated by altering and harnessing the physiological culturing conditions [86]. Compounds isolated from algae, including carotenoids, phycobilins, fatty acids, polysaccharides, vitamins, sterols, polyphenols, lipids, or proteins, have demonstrated antioxidant, anti-aging, photo-protective and anti-inflammatory activities [87] (Table 2).

**Table 2:** Chief cosmetic ingredients from algae

Class	Cosmetic ingredient	Cosmetic application
Chlorophytes	β-carotene	Anti-aging creams and UV protectant Sun-screen lotions
	Lutein	
	Neoxanthin	
	Violaxanthin	
Phaeophytes	Potassium Alginate	Skin care serum and hair gels
	Phlorotannins	Anti-aging creams
	Phloroglucinol	UV protection, skin whitening
	Fucoidan	Sun-screen lotions
	Fucoxanthin	De-pigmenting agent
	Laminarin	Skin protecting cream
Rhodophytes	Carrageenan	Thickening and moisturizing agent in skin cream
	Asthaxanthin	Anti-ageing ointments, lipsticks
	Porphyra-334	Sun-screen lotions
	Shinorin	Sun-screen lotions

### Skin Ageing

Collagen has been validated to slow down skin ageing and enhance the suppleness of skin. Collagen, along with elastin fibers are normally present in skin, helps to maintain youthful, vibrant skin and keeping it flexible and intact after being stretched. The phlorotannins extracts of sea kelp *Eisenia bicyclis* and brown alga *Ecklonia cava* are proved to benefit the skin by reducing the elastase activity significantly [88, 89]. Furthermore, green microalgae such as *Chlorella*, restores the firmness of skin by protecting collagen and elastin fibers against the enzymes, collagenase and elastase which degrades the skin [90] are used as anti-wrinkling agent commercially in skin cream (Dermochlorella^®^). Astaxanthin found in *Haematococcus pluvialis* is remarkably known to regenerate skin tone, elasticity and retain the moisture content of corneocyte layer, thereby resulting in lowering skin wrinkles [91]. Hexadecatetraenoic acid, obtained from Antartic Sea ice diatom, *Stauroneis amphioxys* and Hexadecapentaenioic acid from marine green microalga *Anadyomene stellata* are used in cosmetic preparation for preventing wrinkling, sagging, anti-aging and boosting collagen deposition [92]. The brown algal extract of *Macrocystis pyrifera* is known to induce the hyaluronic acid synthesis, by promoting the synthesis of syndecan-4 in extracellular matrix of dermal tissues [93]. Algae oil based nano-emulsion has demonstrated to inhibit the ramifications of UVA-induced skin impairment by extenuating epidermal water depletion, skin inflammation, and development of melanocytes [94] (Table 3).

**Table 3:** Origin, function of potential biomolecules from algae

	Species name	Type	Compound	Function	References
1	*Alaria esculenta*	Macroalgae	Lipophilic extract	Reduce progerin in aged fibroblast	[93]
2	*Fucus vesiculosus*	Macroalgae	Laminaran, fucoidan and alginate	Increasing expression integrin molecules	[95]
3	*Turbinaria conoides*	Macroalgae	Laminaran, fucoidan and alginate	Antioxidant	[59]
4	*Porphyra umbilicalis*	Macroalgae	Mycosporine-like amino acids	Photo-protective potential	[96]
5	*Synechocystis, Nostoc, Gloeocapsa, Gloeocapsopsis, Scytonema*	Microalgae	Mycosporine-like amino acids	Photo-protective potential	[97]
6	*Gracilaria chilensis,* *Scytosyphon lomentaria Macrocystis pyrifera,* *Callophyllis concepcionensis, Ulva* sp. *and Enteromorpha* sp.	Macroalgae	Polyphenols	Macromolecular antioxidants	[98]
7	*Ecklonia stolonifera*	Macroalgae	Phlorotannins, Oxylipins	Matrix metalloproteinase (MMPs) inhibition activity	[99]
8	*Dunaliella salina, Chlorella species*	Microalgae	Beta-carotene	Photo-protective potential against UV, Anti-oxidant	[100, 101]
9	*Muriellopsis* sp.*, Chlorella zofingensis, Scenedesmus* sp. *and Chlorella* *protothecoides*	Microalgae	Lutein	Anti-oxidant	[102, 103]
10	*Haematococcus pluvialis*	Microalgae	Astaxanthin	Potent antioxidant and scavenger of free radicals	[91]
11	*Chlorella* sp.	Microalgae	Sporopollenin	Anti-wrinkle potential	[87]
12	*Scytonema* sp.*, Lyngbya aestuarii*	Microalgae	Scytonemin	UV-A sunscreen	[104, 105]
13	*Sargassum macrocarpum*	Macroalgae	Sargafuran	Anti-acne activity against P*ropionibacterium. Acnes*	[106]
14	*Spirulina platensis*	Microalgae	Crude extract in skin cream	Wound healing effect of keratinocyte cell	[107]

### UV-Photoprotective and Anti-photoaging Compounds

Algae are recognized to produce a range of UV-protective compounds, like mycosporine-like amino acids (MAAs), phycobiliproteins, flavonoids [108], carotenoids [100], scytonemin [105] and several other photo-protective compounds. MAAs are found in most of algal and cyanobacterial species and are preferred as UV photo-protective compounds. MAAs are known to absorb UV light between 300–365 nm and have high molar coefficient [87]. These properties help them to absorb the UV light more efficiently and scatter the radiation, without generating free radicals. Furthermore, members belonging to the Rhodophyceae (red algae), Ochrophyta, Phaeophyceae (brown algae), and Chlorophyceae (green algae), were exposed to high intense solar rays, demonstrated the accumulation a high level of MAAs, which acts as a protective sunscreen and prevent desiccation of cells. These MAAs are widely employed in sunscreen lotion and creams such as Helioguard 365™ (Mibelle AG Biochemistry, Switzerland) and Helionori™ (Biosil Technologies, France). Algal derived flavonoids such as anthocyanins are effectively validated as an alternative skin care treatment for treating radiation dermatitis during radiation therapy [69].

### Skin-Lightening and De-pigmenting Agents

Melanin pigment imparts the color to the skin and plays an essential role in shielding the skin from damaging effect of UV light and prevents the carcinogenesis. However, overproduction of melanocytes causes hyper-pigmentation or skin darkening. Furthermore, as ageing occurs, the regulation, control and distribution of melanocytes becomes irregular, causing appearance of dark and discolored spots on the skin. Tyrosinase is one of the prominent enzyme for melanin synthesis; inhibiting tyrosinase is an effective strategy to reduce hyperpigmentation. The tyrosinase inhibiting activity of compounds phlorotannin and 7-phloroeckol extract obtained marine brown seaweed, *Ecklonia cava* has been validated to reduce melanogenesis and can be used as potential skin-whitening agent [109]. Asthaxanthin belonging to carotenoids family obtained from *H. pluvialis* are also well known to decreases melanin by 40% in trans-epidermal cells. This helps to defend skin from flakes, reduce age spots, blemishes, and thus making asthaxanthin an attractive component in sunscreen and after sun lotions [87]. Zeaxanthin, obtained from microalgae *Nannochloropsis oculata,* are validated to exhibit anti-tyrosinase activity and can be exploited for skin whitening treatment [110]. The fucoidan present in *Undaria pinnatifida* and *Fucus vesiculosus* extract, demonstrated an antioxidant activity and improved efficacy in skin brightening application, spot reduction and skin protection in topological application studies [111].

### Anti-oxidants

The ageing processes in dermal cells are accelerated due to increase in lipid peroxidation, caused by superoxide anion, OH^−^ radicals and H_2_O_2_ [87]. The anti-oxidants compounds play a vital role in shielding the human dermal tissues from the scavenging effects of such free radicals. Among algae, the rhodophyceae members are predominantly acknowledged for their anti-oxidant potential owing to abundantly presence of colored pigments such as phycoerythrin, phycocyanin and allophycocyanins, carotenoids and xanthophylls [112]. These algal pigments have been commercially exploited in many cosmetic applications such as Pure Clay Red Algae Mask^®^ by L’Oréal Paris. The free radical in skin cells generated due to photo-oxidation is readily quenched by cyanobacterial MAAs such as scytonemin, asterina-330, shinorine, and palythine [105]. *β*-carotene is another potential antioxidant produced by *Dunaliella salina* and is converted to vitamin A, which is essential for good vision, healthy skin and maintenance of mucous membrane [100].

### Hair Care

The commonly used ingredient Sericin protein in hair conditioning and skin formulations, is generally produced by *Bombix mori* (Silk worm), and can also alternatively obtained from microalgae *Chlorella vulgaris* and *Arthrospira platensis* [113]. 7-phloroeckol are validated to promote stimulation of hair growth in dermal papilla cells (DPCs) and outer root sheath cells (ORS) [114]. Algal oil rich in omega-3 is known for its ability to reduce dry and brittle hair, scratchy and itchy scalp, decrease dandruff, and hair fall. Microalgae derived Docosahexaenoic Acid (DHA) and Eicosapentaenoic acid (EPA), regularly used in hair oils, hair serum, hair gels and spray, provide deep nourishment to the hair follicles and scalp to make the hair strong and healthier.

## Conclusion

The rising trend to use biological and eco-friendly products, have augmented a sharp demand of such products in cosmetic Industry. The cosmetic manufacturing companies are continuously placing efforts to extract and use such microbial compounds on industrial scale. The advances in biotechnology, genetic improvement of organism, and immense microbial biodiversity has considerably boosted the use of novel biologically derived compounds in cosmetics. Cosmetics essentially requires interaction and penetration to multilayers of skin and different cell types hence biosufactants, anti-oxidants, anti-aging etc. property compounds produced by microbial sources serves best replacement to chemical entities available in marker. Some of these biologically derived products may cause adverse effect, hence methodical and systematic rigorous assessment by means of clinical research is required to understand the true potential before any validation.
